# Language Development across the Life Span: A Neuropsychological/Neuroimaging Perspective

**DOI:** 10.1155/2014/585237

**Published:** 2014-12-18

**Authors:** Mónica Rosselli, Alfredo Ardila, Esmeralda Matute, Idaly Vélez-Uribe

**Affiliations:** ^1^Department of Psychology, Florida Atlantic University, 3200 College Avenue, Davie, FL 33314, USA; ^2^Florida International University, Miami, FL, USA; ^3^Instituto de Neurociencias, Universidad de Guadalajara, Guadalajara, JAL, Mexico; ^4^Florida Atlantic University, Davie, FL, USA

## Abstract

Language development has been correlated with specific changes in brain development. The aim of this paper is to analyze the linguistic-brain associations that occur from birth through senescence. Findings from the neuropsychological and neuroimaging literature are reviewed, and the relationship of language changes observable in human development and the corresponding brain maturation processes across age groups are examined. Two major dimensions of language development are highlighted: naming (considered a major measure of lexical knowledge) and verbal fluency (regarded as a major measure of language production ability). Developmental changes in the brain lateralization of language are discussed, emphasizing that in early life there is an increase in functional brain asymmetry for language, but that this asymmetry changes over time, and that changes in the volume of gray and white matter are age-sensitive. The effects of certain specific variables, such as gender, level of education, and bilingualism are also analyzed. General conclusions are presented and directions for future research are suggested.

## 1. Introduction

Human language is a communication system in which, via a limited number of meaningless sounds (phonemes), it becomes possible to make a virtually unlimited number of combinations that produce meaningful elements (morphemes, words), which can then be combined to generate an almost endless number of sentences. This property is usually known as the “double articulation of language” [1], which means that the speech stream can be divided into meaningful elements: words that can be further subdivided into meaningless sounds or phonemes. Language structure is characterized by the existence of several levels of analysis [2]. One common distinction is that established in relation to the transmission of meaning via lexicon (vocabulary) and grammar (morphosyntax) [3]. Bickerton [4] emphasizes that symbolic units (lexicon) and syntax (grammar) are the only real novelties in human communication and the most salient of all elements in any adequate theory of language, while Chomsky [5] has made a similar distinction when referring to the conceptual (lexical) and computational (syntactic) aspects of language.

In most adults, language has a well-defined cerebral organization in the left hemisphere that includes two main language systems. The first one, involved in lexical/semantic analysis, is associated with Wernicke's area, while the second, located in the left posterior frontal lobe (Broca's area), is related to grammar (morphosyntax) and speech automatization (i.e., speech praxis) [3, 6]. This organization of language in the brain is not exactly the same in children and older adults, and some significant developmental changes have been well documented. The purpose of this paper is to analyze language development and the changes that occur in its brain organization from birth through senescence, passing through the stages of infancy, childhood, adolescence, and adulthood. It includes findings from both developmental and adult studies, particularly those of interest to neuropsychology and the neuroimaging literature. A comprehensive picture of age-related changes in the volume of gray and white matter is provided by structural magnetic resonance imaging (MRI) studies, while functional MRI (fMRI) and magnetoelectroencephalographic (MEG) methods have generated information on neural activity associated with cognitive functions. Use of blood oxygenation level-dependent (BOLD) signal with fMRI may produce acceptable spatial resolution, and the magnetic fields changes utilized in MEG allow tracking of the neural activity with reasonable time resolution. More recently, the analysis of structural connectivity with diffusion tensor imaging (DTI) (white matter wiring) has given anatomical support to functional brain models of cognition [7]. This technique allows the visualization of the rate and shape of diffusion of water along axons and is used to depict axonal pathways. Thus, the objective of this paper is to integrate age-related changes in linguistic skills to age-related neuroimaging findings.

The first section presents a review of the development of language functions (phonology, vocabulary, grammar) during infancy and the preschool and school years, before narrowing the discussion to the development of specific language skills, such as confrontation naming (CN) (considered a major measure of lexical knowledge) and verbal fluency (VF) (regarded as a major measure of language production ability). Language evolution in adults and changes during senescence are analyzed next. Neuroimaging findings related to language development are introduced in each section. The influence of such additional variables as gender, level of education, and language experience on language development is highlighted at the end of the paper. Note that this review focuses on the development of oral language and does not include written language.

## 2. Language Development in Infancy and the Preschool Years

It has been well established that newborns respond to auditory stimuli in the range of language frequencies and show an overt preference for verbal sounds [8, 9], suggesting a biological predisposition to detect and process human language signals. From 2 to 8 months, babies demonstrate an evident orientation to verbal sounds that gives rise to the so-called “mother/father-child dialogue.” Using the habituation paradigm (in which infants eventually lose interest in a repeated stimulus and cease to respond to it), it has been shown that babies aged 22 to 140 days are capable of detecting consonant-vowel (CV) changes much better in the right ear (left hemisphere) than the left one (right hemisphere), a finding which indicates that the left hemisphere is likely involved in processing language-related signals right from birth [10]. This is a particularly important finding because it suggests an inborn brain asymmetry for language.

The results of neuroimaging studies are congruent with the above observation, as they have shown that very early in life human language is predominantly processed by the left hemisphere. Thus, Dehaene-Lambertz et al. [11] used fMRI to demonstrate that breastfed babies activate restricted perisylvian brain areas in the left hemisphere when listening to their mothers' language. These brain areas are similar to those involved in language in adult brains (e.g., Wernicke's area in the left hemisphere). This suggests that the infant's cerebral cortex is already structured into various functional regions, one of which is active in language reception. In newborns, as in adults,* listening to speech* activates a large subset of temporal lobe areas with a marked left-hemispheric dominance.

The issue of language lateralization towards the left hemisphere from birth, however, is not universally supported, as some authors (e.g., Dick et al. [12]) have departed from the electrophysiological literature, questioned the exclusively innate cerebral organization of language, and postulated a more dynamic developmental process. They argue that although event-related potential (ERP) components of auditory stimuli show early left lateralization (from 3 months to 3 years), symmetrical cerebral distribution is seen later in life, from 6 to 12 years. Also, connectivity during language listening evolves from interhemispheric connectivity in infants to the predominant connectivity in the left hemisphere during adulthood. While the classic language regions are activated by the age of 6, the functional connectivity among these regions is not. Unlike adults, who show robust connectivity between the frontal and temporal language regions in the left hemisphere, the language network in children is characterized by a strong functional interhemispheric connectivity, mainly among superior temporal regions, as revealed by low frequency data from fMRI experiments on language processing [13]. The asymmetric organization of language, examined with fMRI and neuropsychological tests, increases between the ages of 8–20 years [14]; therefore, early lateralization of language must be understood in the dynamic context of changes in brain activation that take place over the entire life span, a context in which experience plays a particularly relevant role.

Consequently, it is important not only to consider biological variables when analyzing brain organization and the lateralization of language but also to include interaction with environmental conditions. It could be conjectured that the brain mechanisms required for language are not fixed at birth but present a dynamic organization during their development and exposure to language [15].

The child's experiences may play a significant role in this language lateralization process. For instance, the influence of environmental variables on the cerebral functioning of language is evident in the phenomenon called “perceptual narrowing,” in which perception is broad at birth, but narrows as a function of experience [16], such that while at birth babies are endowed with universal recognition of phonemes (native and non-native), by the end of the first year a clear decline in the recognition of nonnative phonemes (i.e., those to which they are not exposed) is observed [17, 18]. Two theories have been offered to account for the phenomenon of perceptual narrowing. First, the so-called regressive theories of neural development propose explaining it on the basis of the selective elimination of certain connections (known as pruning) [19]. Other authors, in contrast, propose that the perceptual narrowing observed at the functional level is likely due to the formation of new connections (called selective elaboration of synapses) [20]. At present, we lack sufficient evidence to determine which one of these neurofunctional explanations is correct or whether the two are contradictory or complementary.

### 2.1. Phonology

Phoneme production in the native language seems to increase parallel to the growing perceptual sensitivity to environmentally relevant phonemic distinctions (native language phonology) and decreasing sensitivity to environmentally absent distinctions; that is, perceptual narrowing occurs. Tables 1 and 2 show the acquisition of consonant phonemes in children whose native tongue is English or Spanish. There, 90% of the children exposed to the English language from birth were able to produce 5 consonant phonemes by age 3, 4 more phonemes by age 4, and the complete phonological repertoire by age 8 [21, 22]. Interestingly, the acquisition of Spanish phonemes seems to occur more quickly, as 90% of the children exposed to that language acquired all but 3 of its phonemes by age 4, while by age 6 they had completed the acquisition of the entire range [23, 24]. This apparent difference in phonemic development between English and Spanish can probably be attributed to two main sources: (1) these studies focused only on the production of consonants (no vowels, see Tables 1 and 2) and (2) English has more phonemes (about 34) than Spanish (about 23).

We know that phonological abilities develop in a way that corresponds to the brain's growing specialization in terms of recognizing native language phonemes [25]. During the second and third years of life, the ability to not only perceive but actually produce native speech sounds increases significantly, so that by the age of 4-5 years phoneme repertory development doubles, and in the range of 6-to-8 years the typical child's phonological repertoire is complete, regardless of her/his phonological language system [22, 26].

In a meta-analysis of the brain/language fMRI literature conducted by Vigneau et al. [27], phonological processing activation peaks were found in the left frontal lobe and the left temporal and inferior parietal areas. Among the phonological tasks included in the studies reviewed were syllable repetition or articulation, reading, listening or attending syllables or letters, reading a pseudo-word or counting the number of syllables it contains, counting the syllables in a word, and discriminating whether trial words ended with the same sound.

### 2.2. Vocabulary

Active vocabulary normally begins to develop early in the second year of life. Most children produce their first recognizable words between 12 and 18 months of age. After the first year, word comprehension begins to increase rapidly, though at this age a clear dissociation exists between language expression and comprehension; that is, children's ability to understand language significantly surpasses their capacity to produce it [28].

**Figure 1:**
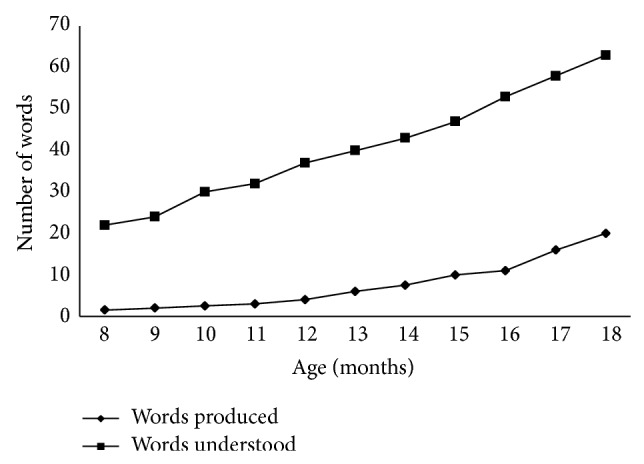
Number of words produced and understood by Spanish speakers in the 50th percentile according to the Spanish-language MacArthur-Bates Communicative Development Inventories Short Form I (S-CDI SFI) and Spanish-language MacArthur-Bates Communicative Development Inventories Short Form II (S-CDI SFII) (adapted from Jackson-Maldonado et al. [29]).

The developmental discrepancy between word comprehension and word production in Spanish-speaking toddlers was reported recently by Jackson-Maldonado et al. [29] and is shown in Figure 1. By the age of 12 months, children in the 50th percentile produced fewer than 10 words but understood close to 40. At 18 months, this gap persists; that is, while word production doubles to almost 20, comprehension reaches 60. It is interesting to note that similar findings have been reported for English-speaking toddlers [28].

From 18 to 30 months there is an important increase in vocabulary size and in the comprehension of words that are presented out of context. Also, toddlers in this age group begin to combine words to create sentences and to use language to ask for information. Thus, the increase in vocabulary size correlates with an increase in grammar complexity [30].

According to Fenson et al. [31] and Lorraine [32], by the end of the first year of life children have mastered perhaps 20 words, but by age 2 their vocabulary will have grown tenfold, and by age 3 the child will have close to 1000 words, a number that will double by age 5. At 6 years of age, the number of words averages 2,600, but the child's comprehension includes approximately 20,000 words, a level of understanding that will double again by age 12.

The tremendous speed of language development observed by age 2 has been linked to structural changes in the neurons (such as the growth of axons and a larger number of dendrites) and upsurges in the myelination process that permit faster conduction. A normal newborn has only sparse neural circuitry, but as age increases there is a tremendous expansion in the complexity of those circuits that is reflected in the marked increase in the number of dendrite arbors from birth to 2 years [33]. In addition to more general changes in the neuronal structure, Su et al. [34] used quantitative analysis of MRI images to assess myelination-associated developmental changes in the signal intensity of language-correlated regions in infants and children. Those authors found that myelination in the classic language areas, that is, Broca's and Wernicke's areas, reaches mature appearance by 18 months, which coincides with the age at which children begin to actively produce language and initiate grammatical development. Pujol and colleagues [35] used three-dimensional MRI to quantify myelination in the lateral part of the left hemisphere from birth to 3 years and found that it begins to increase in the sensorimotor white matter and the Heschl gyrus (primary auditory area) and later extends into the aforementioned language-related areas. These authors suggest a process of simultaneous maturation of the temporofrontal language network, since both comprehension and production regions showed very similar myelination progress during the first 3 years of life.

Leroy and colleagues [36] quantified the degree of maturation in the linguistic network in fourteen 1-to-4-month-old infants using MRI spatial resolution and found that the least mature perisylvian region was the ventral superior temporal sulcus (STS). They also observed a significant difference in maturation in the STS that favored the right side, which they interpreted as an early indication of the distinctive left-right development of this structure. Asymmetries in the maturation of Broca's area correlated with asymmetries in the frontotemporal dorsal pathway might provide infants with a phonological loop circuitry much earlier than was previously assumed.

### 2.3. Grammar

Even before children begin to speak, they can detect complex linguistic cues from auditory input, including structural regularities [37]. Between 2 and 5 years of age, the learning of morphosyntactic rules in simple sentences can be detected, together with the onset of the construction of progressively more complex sentences [38]. Grammar develops rapidly during this age range with a significant increase in average phrase length from 2.0 to 4.5 words [12].

A reliable index of language acquisition in young children and one that is described conventionally is mean length of utterance (MLU), which corresponds to the number of words in a sentence (MLUw) or the number of morphemes (MLUm) used in spontaneous conversations. Rice et al. [39] reported the MLUw and MLUm of 136 monolingual, English-speaking children ranging in age from 2 years 6 months to 8 years 11 months.  Table 3 presents their results by year range. As can be seen, the MLUw and MLUm by age range are closely aligned; that is, children advance from producing an average of 3 words, or morphemes, per utterance at age 2, to 5 words or morphemes per utterance by age 8. Similar results have been reported for the extension of utterances in normal Spanish-speaking children [40].

Although brain lateralization of language begins early on, improvement in language abilities (including syntactic ones) is associated with an increased lateralization of language functions in the left hemisphere [7]. For example, between 7 and 12 years of age, better syntactic skills are related to an increase in left inferior frontal gyrus activation and a decrease in right inferior frontal activation as measured by fMRI [41]. Also, significant increases in the left frontal lateralization for verb generation with advancing age beginning at age 5 have been reported using magnetoencephalography [42].

## 3. Language Development during the School Years

The period in which children begin school (around age 6) is considered critical for their cognitive development. During this time, teaching at school awakens knowledge of the components of language at all levels of analysis: phonological, lexical, semantic, grammatical, and pragmatic. Development of such knowledge is intimately related to cognitive evolution and is associated with progress towards the stage of concrete operations. The introduction into the world of formal instruction enriches and modifies the linguistic input to which a child is exposed, such that the drive towards linguistic reflection permits the development of metalinguistic understanding [43].

By age 6, children present well-developed language skills. They possess a basic vocabulary of close to 3,000 words, virtually complete phonological production ability (i.e., they can produce all the phonemes and phoneme combinations of the mother tongue), and can correctly understand and use basic grammar [22].

From the age of 6 years to puberty (around 12), strategies for generating and integrating information emerge, as does the use of unusual sentences (sophistication of language grammar). Lexicon continues to increase in an enhancement that correlates significantly with more advanced levels of schooling. A progressive increase of metalinguistic awareness is also found that is due, in part, to the development of reading skills [44].

MRI neuroimaging studies have demonstrated increases of white matter (WM) volume throughout childhood and adolescence [45], which may underlie a greater connectivity and integration of incongruent neural circuitry [46]. Though this rapid increase in the volume of WM takes place in both hemispheres, a more significant increase in the left language-associated regions (frontotemporal) has been reported in children and adolescents using computational analysis of structural MRI [47]. Wilke et al. [48] found that while listening to a story children between the ages of 6 and 15 years present bilateral activation of the language regions (superior temporal, inferior parietal, and inferior frontal brain, in an fMRI paradigm) with leftward dominance. Clearly, language development at these ages is linked to the development of other, nonlinguistic abilities, such as attention, social skills, memory, and many other individual characteristics [26]. More talkative children, for example, may have the opportunity to practice more language skills through increased verbal interaction. Also, children with larger verbal memory capacity may repeat longer sentences, retain more words, and so develop a larger vocabulary. It is reasonable to think that the development of language areas in the brain occurs parallel to the maturation of other brain areas and parallel to the increased connectivity between the temporal and frontal lobes (language areas) and other brain structures (e.g., the hippocampus) that comes with higher age. In fact, DTI studies have demonstrated that the integrity (measure by FA values) of most major WM tracks increased with age during childhood and early adulthood [49] and that temporal lobe gray-matter structures (the amygdala and hippocampus) seem to increase in volume during childhood and adolescence [50].

The transition from childhood to adolescence is characterized by both structural and functional brain changes. The total cerebral white matter proportion in a structural MRI study is significantly greater than the change in the total cerebral gray matter proportion [51], while the reduction in gray matter correlates significantly with increases in white matter [52]. The few studies that have analyzed the association between these anatomical changes and cognitive performance during adolescence have found better performance associated with white matter diffusion properties [53, 54]. Meanwhile, fMRI studies comparing the trajectory from childhood to adolescence have shown changes in brain activation during language production tasks (speaking) from bilateral towards increasingly lateralized representation in the prefrontal cortex (premotor areas) [55].

### 3.1. Development of Confrontation Naming (CN) and Verbal Fluency (VF)

Specific neuropsychological tests have been widely used with children and adolescents to measure cognitive development and diagnose language disorders. Particularly influential in this regard are two tests: CN (finding figure names), and VF (saying words that correspond to a semantic category (semantic condition) or that begin with a particular phoneme (phonemic condition)), which are useful diagnostic tools that can effectively identify word finding and language production defects in diverse neuropsychological conditions.

Riva et al. [43] conducted a study with 160 participants divided into 5 groups according to the school grade they were attending: from 1st to 5th. Results revealed consistent improvements in performance by grade, with higher scores on semantic fluency tasks than phonemic fluency tasks at every point. These findings provide support for the claim that these two tests reflect different abilities and, therefore, depend on distinct cognitive domains and brain networks. In the Boston Naming Test (BNT) (an often-used neuropsychological measure of lexical knowledge), participants increased the number of correct answers as age and years of schooling increased. No gender or year of schooling × gender interaction effects were found. Correlation analysis revealed closer correlations between the BNT and semantic fluency tests than with the phonemic fluency test, as the latter proved more difficult than the former in all groups tested. The authors hypothesized that this may be because the specific ability demanded by the phonemic condition depends on the maturation of the frontal system and, hence, the development of executive functions.

In her study of a sample of Hebrew-speaking children, Kavé [56] determined that only certain naming and fluency measures reach adult levels during adolescence. As children develop, their naming test performance improves until reaching adult levels at age 16 to 17. However, 16-to-17-year-old subjects name fewer items spontaneously and require more functional cues to arrive at the correct answer than do adults aged 18 to 29 years, suggesting not only that the vocabulary required to successfully complete the naming test has been acquired by age 16 to 17 but also that maturation of strategic retrieval functions may still be lacking.

In a study conducted with a sample of monolingual Spanish-speakers made up of 171 children divided into 5 age groups (6-7, 8-9, 10-11, 12-13, and 14-15 years), Matute et al. [57] found that at age 6-7 children can generate about 10 animal names in one minute; by age 8-9, about 11; by age 10-11, about 12; by age 12-13, about 13; and by age 14-15, about 16. Phonemic fluency increased on average from about 3.5 at the age of 6-7 to around 13 at 14-15 years. While all fluency test scores increased from age 6 to 15, the most significant changes were seen after age 12-13, a finding consistent with the hypothesis that they depend on the maturation of executive functions. It is important to note that this is the age at which brain activation patterns during verbal generation are lateralized in the left hemisphere [58].

Sauzéon et al. [59], meanwhile, measured clusters that consisted of successively generated words belonging to the same semantic category (for instance, animal names that refer to pets or to zoo animals, etc.) or phonemic subcategory (for instance, words beginning with /a/ to say animal names or fruit names, etc.). Cluster size (i.e., number of elements per subcategory) was counted from the second word of each group and switches were calculated as the number of times a subject changed from one cluster to another. The number of switches increased from 11 to 12 years on the phonemic fluency test but decreased with age on the semantic task. The authors of this study hypothesized that late frontal network maturation may explain why greater changes occurred on the phonemic fluency test with regard to the number of both switches and clusters, considering that this is, in part, an executive function test. They attributed the increase in cluster size seen over the course of the development of semantic fluency to the enrichment of semantic knowledge. Thus, continuous vocabulary expansion may be responsible for the fact that adults generate more words than teenagers.

The use of clustering strategies in semantic and phonemic fluency was also tested in the 3rd- and 5th-grade children (aged 8-9 and 10-11 years, resp.) by Koren et al. [60]. Consistent with the results outlined above, semantic fluency was greater than phonological fluency in both age groups. Also, the 5th-grade children had greater semantic and phonemic fluency than those in the 3rd grade, a finding associated with an increase in the number of clusters but not cluster size. This increase, and the related increase in fluency in older children might thus be related to the development of cognitive flexibility. See Table 4 for a summary of these studies.

### 3.2. Patterns of Brain Maturation

**Figure 2:**
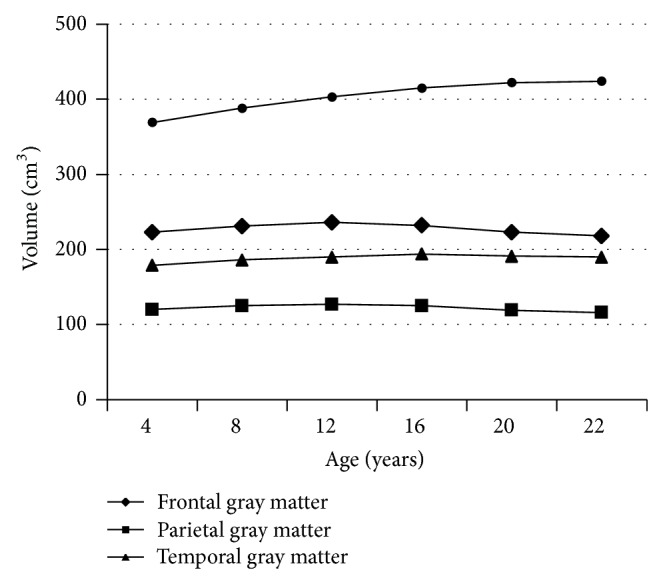
Changes in gray and white brain matter between the ages of 4 and 22 years in males (adapted from Lenroot et al. [61]).

Verbal generation measured by VF tests and vocabulary size measured by naming tests are obviously correlated with some of the neuroanatomical and neurophysiological changes that occur in the brain during childhood and adolescence. Functional and structural MRI studies have shown that one of the most important aspects of maturation across the cerebral cortex after age 5 is the overall decrease in gray matter (GM) volume and the continuous increase in the volume of white matter (WM) [61]. The development of GM follows an inverted U pattern, with initial growth followed by a continuous decrease [62, 63]. The age at which this decrease in GM begins varies across the cerebral cortex; for example, the frontal system reaches its GM peak between the ages of 12–14 years, while in the temporal lobe this occurs around age 17-18, and in the parietal at 10–12 years. In contrast, the total volume of WM increases continuously (see Figure 2). Giorgio et al. [52] used diffusion-weighted magnetic resonance imaging to test for age-related WM changes in 42 adolescents (aged 13.5–21 years). They found that the increase of WM is much more prominent than the decrease in GM, results which revealed that the most significant changes were in the body of the corpus callosum (related to the integration of sensory and motor cortical information) and the right superior region of the corona radiata (fibers projecting to and from the entire cerebral cortex, particularly the motor cortices).

Findings from imaging studies suggest that age-related WM changes continue beyond early childhood. Myelinated fibers are the presumed substrate for greater brain connectivity, for acquiring new abilities, and for increases in learning [46, 64]. The volume of most brain tracts using diffusion tensor tractography shows a significant increase between childhood and adolescence, with volume increases still being evident in several association cortex tracks during the postadolescent years [65]. Furthermore, gender differences in the maturation rate of both gray and white matter have been reported, with boys showing a faster rate of change than girls [62].

In addition to general changes in brain volume and gray matter, increments and decrements in the activation of specific brain regions have also been associated with language development. For example, Brown et al. [66] explored progressive and regressive developmental changes in the functional brain organization that underlies lexical control in 95 healthy individuals aged 7–32 years. They used event-related functional magnetic resonance imaging to identify those brain regions that revealed statistically reliable, age-related effects. These brain regions were divided according to whether adults or children showed greater activity. Their results show that 75% of the regions studied (30/40) manifested decreases in activity as age rose. The areas marked by developmental decreases were distributed bilaterally and were evident most prominently in the medial-frontal and anterior cingulate cortex, the right frontal cortex, the medial-parietal and posterior cingulate cortex, and the bilateral occipitoparietal cortex. In contrast, only 25% (10/40) of the age-related brain regions demonstrated increases in activity as age increased. Most of the regions that showed significant developmental increases were in the left lateral and medial dorsal frontal cortex and the left parietal cortex, including the supramarginal gyrus. The brain regions that expanded and those that contracted showed signs of becoming adult-like at different ages. The activity in decreasing, age-related regions on average became 50% adult-like at age 12.8 years and 75% adult-like at age 16.5. Regions showing maturational increases, on the other hand, matured somewhat earlier, showing peak activity that was 50% adult-like by the age of 11.9 years and 75% adult-like by age 14.8. In summary, performance on word generation tasks appears to be related to increases in the activation of the left frontal and parietal cortex that reaches a peak around age 13 and to maturational decreases in other brain regions that achieve an adult-like condition between the ages of 13 and 16 years.

Szaflarski et al. [67] used a longitudinal design to obtain additional evidence for progressive and regressive changes in brain development during the school years. They obtained fMRI data annually for a period of 5 years using a verbal generation task paradigm. Results demonstrated a progressive participation in language processing by the inferior/middle frontal, middle temporal, and angular gyri of the left hemisphere and the lingual and inferior temporal gyri of the right hemisphere, accompanied by a regression in the participation of the left posterior insula/extrastriate cortex, the left superior frontal and right anterior cingulate gyri, and the left thalamus. These authors suggest that the development of language representation in the brain reflects qualitative rather than simply quantitative changes and concluded that their results provide evidence of the increased neuroplasticity of language in this age group.

### 3.3. Development of Brain Lateralization

To pinpoint the significance of brain lateralization values, the so-called “lateralization index” has been proposed (e.g., [58, 68]) that employs fMRI-activation during performance of language tasks. This index refers to the difference between the number of activated pixels in the left (L) and right (R) hemispheres divided by the total number of activated pixels. Analyses of the lateralization of different functions have shown that one of the cognitive functions with the highest lateralization indexes in the left hemisphere is language. Though a certain degree of functional lateralization has been observed in the human brain from birth, the assumption that lateralization increases with age means that the lateralization index can be used as a measure of brain maturation (e.g., [69]).

The increased lateralization of language in the left hemisphere as age advances has been correlated with the growth of the corpus callosum, which connects the associative cortex of the two cerebral hemispheres and expands significantly from 2 to 15 years of age [70]. The anterior regions of the corpus callosum mature first (at 3–6 years), followed by growth in the posterior ones (isthmus and splenium) as shown in [71]. Using time series of three-dimensional magnetic resonance imaging scans, Westerhausen and colleagues [72] showed that children aged 6–8 years whose callosal isthmus increased in thickness over the course of 2 years showed a decrease in interhemispheric information transfer, whereas children who exhibited a decrease in isthmus thickness showed an increase in information transfer. These findings support the notion of a relation between the structural and functional development of the corpus callosum. Moreover, the authors suggest that the refinement of the connections of this commissure that occur after age 6 optimize neural communication between the two cerebral hemispheres.

## 4. Language Evolution in Adults and the Elderly

In the same way that language production and comprehension can reveal brain development in the early stages of human life, language abilities continue to reflect cerebral changes throughout adulthood and into senescence. Although verbal abilities are relatively less sensitive to the aging effect compared to nonverbal skills, some age effects on the latter are still observable. For example, Brown [73] reported that the “tip-of-the-tongue” phenomenon increases with age, reflecting a certain degree of naming deficit (anomia), while Ardila [74] described decreases in lexical access associated with age as measured by the vocabulary subtest of the Wechsler Adult Intelligence Scale. According to the normalization data of the WAIS-III [75], vocabulary subtest scores increase up to the age of 45–54 years, but a decline is observed after that. More recently, Verhaegen and Poncelet [76] found that subtle naming difficulties, reflected by an increase in naming latencies, appear in individuals as young as those still in their 50s.

Interestingly, lateralization of language seemingly presents some changes during senescence, as greater activation of the right hemisphere during language comprehension and production tasks has been reported among elderly subjects. This observation suggests that the degree of language lateralization decreases after a certain age, while cognitive processes become more symmetrically represented over time [77]. Szaflarski et al. [78], for instance, examined the effect of age on language lateralization in 170 healthy, right-handed children and adults aged 5–67 years using functional MRI (fMRI) and a verb-generation task. They found that language lateralization towards the left hemisphere increases between the ages of 5 and 20 years, levels off between 20 and 25, and slowly declines from 25 to 70.

Recent research describes highly dynamic and plastic cerebral and cognitive systems during aging. Studies using functional neuroimaging have shown that the brains of older adults respond to the cognitive changes characteristic of aging through anatomical and physiological modifications. Cabeza and colleagues [79] have suggested that during cognitive task performance a reorganization of brain activation patterns occurs that is age related. Two activation patterns distinguish older adults from younger ones, as those authors show (1) bilateral activation of the prefrontal lobes in cognitive tasks that in younger adults is lateralized to one hemisphere and (2) a reduction in occipital-temporal activation with increased activation of the frontal areas. These functional brain changes have been unified in models of reduced brain asymmetry in aging, or HAROLD (*hemispheric asymmetry reduction in older adults*) [80], and changes in posterior to anterior activation, or PASA [81]. The decrease in posterior activation and increase in anterior activation in older brains have been interpreted as part of a compensatory strategy by the frontal lobes [82].

It should be pointed out that decreased asymmetry is observed not only in the neocortex but also in other brain areas, including the hippocampus. Maguire and Frith [83] selected 12 young (23–39 years old) and 12 older subjects (67–80) and asked them to retrieve real-life autobiographical event memories accrued over decades. fMRI recording was performed simultaneously. Several commonalities were observed between the younger and older groups in terms of the network of brain areas activated during retrieval. However, while left hippocampal activation was apparent in the younger group, bilateral hippocampal activation was manifested in the older adults. Direct comparisons of the two groups confirmed significantly greater right hippocampal activation in those older adults.

### 4.1. Confrontation Naming and Verbal Fluency in Adults

It has often been assumed that word retrieval difficulties are found commonly in older adults; indeed, several studies have reported evidence supporting an age-related decline in lexical retrieval ability (e.g., [84–86]). However, other research has failed to find evidence of such an age-associated lexical retrieval defect (e.g., [87, 88]). We can conjecture, therefore, that there may be some variability in the decline in lexical retrieval or perhaps that the different experimental approaches using distinct tasks with a variety of study population account for some of the variation in results.

Kent and Luszcz [89], for example, studied an initial sample of 803 people with an average age of 76 (range 65–93) who underwent an initial examination and a follow-up evaluation 2 years later. Finally, a subsample of 326 subjects was reevaluated 6 years after that. Results indicated that age and educational level, but not gender, affected naming ability. The authors concluded that there was a continuous decline in naming ability that correlated inversely with age. Such findings have been observed in both cross-sectional and longitudinal studies. Interestingly, in a 20-year longitudinal study, Connor et al. [90] reported a decline of approximately 2% per decade in BNT scores.

**Figure 3:**
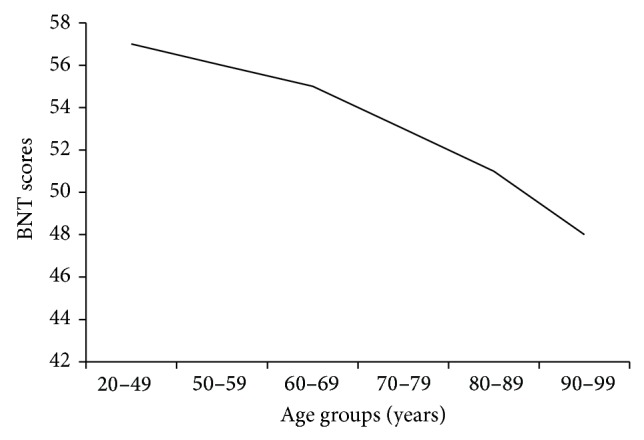
Average Boston naming scores by age groups (adapted from Zec et al. [91]).

Zec et al. [91] meanwhile analyzed performance on the BNT by 1,111 “normal” elderly (ages 50–101) and 61 younger adults (ages 20–49). They found that mean BNT scores decreased but the* standard deviation increased* with each succeeding decade of age. The size of the decline in mean BNT scores also increased with successive age decades; that is, there was an accelerating rate of decline associated with age (see Figure 3). It is important to emphasize that during normal aging a decrease in mean naming scores is observed, coupled with an increase in the standard deviations of the scores, a finding pointed out previously by Ardila [74], who suggests that as age advances people become more and more heterogeneous in terms of cognition. The observed decrease in cognitive test scores and the increase in variability with aging were also reported by Weintraub et al. [92] in a sample of 1,101 healthy volunteer physicians (aged 28–92 years). This observation means that during aging some individuals present a rapid decline in cognition that eventually results in symptoms of dementia, while others maintain high cognitive test performance (“successful aging”). The majority of the population falls somewhere between these two extremes.

It is noteworthy that, when studying language in general and naming ability in particular, most researchers have focused primarily on children and the elderly, frequently leaving a gap that spans adolescence and early adulthood. Kent and Luszcz [89] analyzed 22 cross-sectional studies and one longitudinal study [93] published between 1980 and 2001 on the effects of age, education, and/or gender on BNT performance in younger and older adults. Based on their review, they concluded that there was a continuous decline in naming abilities that correlated inversely with age, since the results of the cross-sectional studies and the longitudinal analysis were similar.

As observed in younger individuals, older participants across age groups also tend to perform better on semantic fluency tasks than phonemic fluency tasks. It is assumed that during a semantic fluency task there is an activation of an entire semantic category that leads to automatic retrieval of semantically related words. The differences in performance between these two tests (semantic versus phonemic fluency) might be explained by the hierarchical organization of the two categories (phonemic versus semantic), since retrieval by letter requires exploring more subsets of categories than does retrieval of a set like animal names, for example [43]. Moreover, performance on semantic category tasks tends to be better because the task itself provides a structure that the phonemic fluency task does not [94]. Phonological fluency requires processing the phonemic characteristics of words according to a given rule (i.e., same first letter or sound), such that phonological fluency tasks demand that subjects make correct selections, inhibit intrusions, and maintain a constant level of focused attention [95]. Semantic fluency is believed to be more automatic, as it relies on common rules of categorization, whereas phonemic tasks rely on higher-order cognitive functions. Indeed, retrieval by letter appears to require exploring more subsets of words than retrieval of examples from a given semantic category [59].  Table 5 presents verbal fluency scores by age group according to different authors from studies of adult populations.

### 4.2. Patterns of Brain Activation in Adults

The patterns of brain activation observed during performance of CN tests have also been analyzed using structural MRI and diffusion tensor imaging (DTI) data, and reports indicate that the volumes of the left mid-frontal gyrus and right middle temporal gyrus correlate with accuracy on the Action Naming Test (which requires naming actions, not figures) [96], while the volumes of the left mid-frontal gyrus and left planum temporale were seen to be negatively correlated with reaction times for correct trials on the BNT (i.e., those with greater volume are, on average, faster). Also, subjects with greater white matter density tended to achieve greater accuracy and faster reaction times. Better naming abilities were associated with the use of the bilateral perisylvian and dorsolateral frontal areas of both hemispheres. The authors of this study suggested that the older adults with relatively better naming ability may be relying on right-hemisphere perisylvian and mid-frontal regions and pathways in conjunction with left-hemisphere perisylvian and mid-frontal regions to achieve better test performance.

In general, fMRI results show relatively consistent areas of activation during VF tasks. Several studies have found that the areas of significant activation are the left prefrontal cortex, including the middle frontal gyrus [97, 98], and the right cerebellum, while areas of decreased activation are reported bilaterally in the mesial and dorsolateral parietal cortex [97]. Activation of regions of the prefrontal cortex is consistent with the demands on executive functioning involved in task performance. The VF paradigm also activates regions of the inferior frontal gyrus known to be involved in word retrieval, phonological processing, and language production, that is, Broca's area [98]. Using a covert verbal fluency task, Amunts et al. [99] found bilateral activations of the posterior part of the frontal cortex including the inferior frontal gyrus, the precentral gyrus, the parietal lobe, the orbitofrontal gyrus/insula, and the cerebellum, with more extensive activations on the left side than the right one.

Different studies report slight variations in the areas of activation, which can be accounted for by variations in how the methods are applied and by individual differences in cognitive strategies. With regard to semantic fluency tests, Meinzer et al. [100] reported that fMRI peak activity during such a task centered on the junction of the superior temporal gyrus and the inferior frontal gyrus, with additional activity found in the left cuneate gyrus and the medial and middle frontal gyri, while activity in the right hemisphere was confined to the caudate nucleus. The pattern of activity during the phonemic fluency task was very similar, though a larger network of brain regions appeared to be activated and peak activity in several regions was more pronounced. In particular, a large anterior cluster was activated in the left hemisphere that included the left superior temporal gyrus and the inferior frontal gyrus. Also, the superior frontal gyrus, the cuneate gyrus, and the caudate nucleus were activated. In CN tasks, increased activation has been observed in the left inferior temporal gyrus (Brodmann areas 19 and 37) and bilaterally in the middle and inferior occipital gyri (Brodmann areas 19 and 18), regions that form part of the occipitotemporal ventral pathway involved in object recognition and the semantic processing of visual information [98].

Abrahams et al. [98] developed two fMRI paradigms to analyze verbal fluency and confrontation naming. They recruited 18 healthy, right-handed participants (14 men, 4 women) for their study. During the verbal fluency task, participants heard an auditory cue of one letter via headphones and had to respond overtly with a word that began with that letter during the 4 s period they were allowed to respond. In the confrontation naming task, subjects were presented with a visual line drawing of an object for 4 s and had to say the correct name of the object during the response period. Verbal fluency was associated with activation in the middle frontal gyrus (Brodmann areas 46 and 9), the anterior cingulate gyrus, and the inferior frontal gyrus (areas 44 and 45), whereas confrontation naming activated areas of the temporal-occipital cortices (areas 18, 19, and 37) and the inferior frontal gyrus. The authors concluded that these two paradigms successfully activated the regions involved in executive (frontal lobe areas associated with the verbal fluency task) and word retrieval processes (temporal-occipital areas in the left hemisphere).

**Figure 4:**
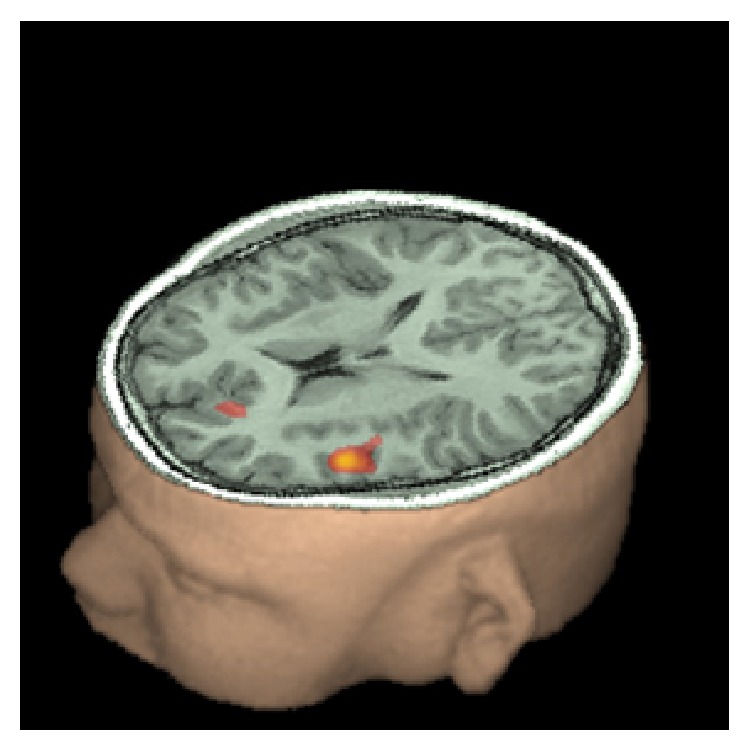
fMRI activation in a right handed 13-year-old boy while performing a verb generation task. Activation of left Broca's area is observed. The small coactivation of the medial frontal cortex is most likely related to selective attention, required during the task. Courtesy Dr. Byron Bernal, Miami Children's Hospital, Radiology Department. Miami, FL, USA.

**Figure 5:**
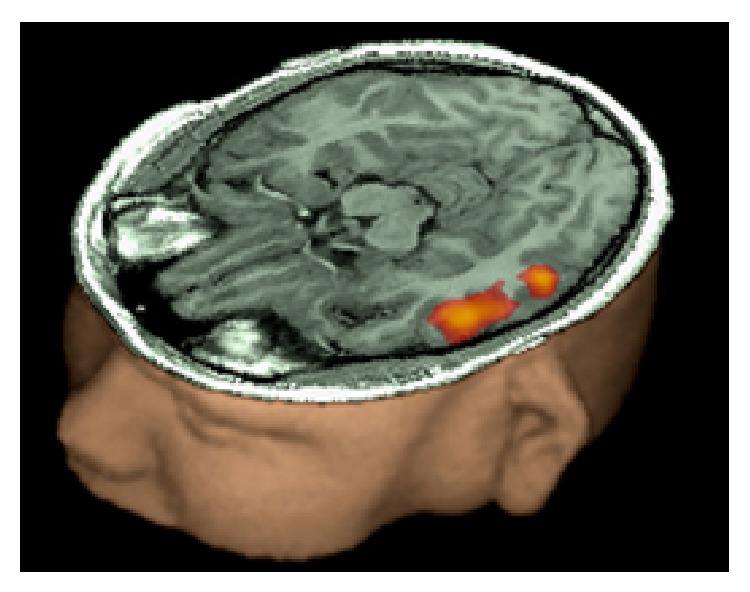
fMRI activation of the left superior temporal lobe (Wernicke's area) during a receptive language task (discriminating antonyms from synonyms) in a right handed 13-year-old boy. Courtesy Dr. Byron Bernal, Miami Children's Hospital, Radiology Department, Miami, FL, USA.

**Figure 6:**
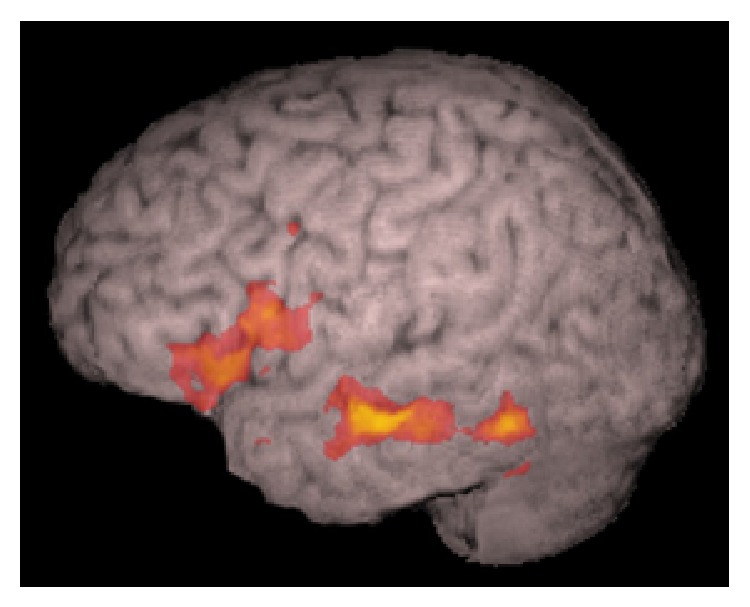
fMRI activation rendered in a 3D brain volume. The left hemisphere is depicted. Activation is seen on the foot of the motor primary area, Broca's and Wernicke's areas during a task involving expressive and receptive language functions (discriminating correctness of sentences describing objects) in a right handed adolescent boy. Courtesy Dr. Byron Bernal, Miami Children's Hospital, Radiology Department, Miami, FL, USA.

Figures 4, 5, and 6 present some examples of fMRI activation during different language tasks.

Briefly, normal adults present greater activation in the left inferior frontal and lateral temporal cortex during both VF and CN. As mentioned above, bilateral activation has been reported in children, but adolescents aged 13 manifest activation of the left hemisphere similar to that of adults when performing VF tasks [58]. It is worth noting that this is the age (around 13 years) at which the most significant improvement in performance on VF tasks is usually seen [57].

One of the areas clearly associated with word production and one that requires special analysis is Broca's area, which corresponds to Brodmann areas 44 and probably also 45, in the left hemisphere. Both VF tasks (semantic and phonemic) involve Broca's area but differ in their participation in semantic processing, as the left Brodmann area 45 is more highly involved in verbal fluency tasks with high semantic load [99]. Regardless of the diversity of functions of Brodmann area 44 ([101] see http://www.fmriconsulting.com/brodmann/Introduction.html), it could be regarded as more of a “motor programming” area, whereas Brodmann area 45 is more of a “language conceptual” area. Damage in Brodmann area 44 (and in the anterior insula) has been associated with speech apraxia [102, 103], whereas pathologies of Brodmann area 45 have been related to extrasylvian (transcortical) motor aphasia [104].

Taken together, all these neuroimaging studies contribute to a better understanding of the neurological bases of language development across the life span [105], particularly the development of word recall as measured by verbal fluency and confrontation naming tasks.  Table 6 presents the main findings of these studies.

## 5. The Effect of Some Specific Variables

As this literature review suggests, age constitutes the essential variable of language changes across the life span and correlates with modifications in brain activation during performance of language tasks. There are, however, other variables that may modulate age effects, among which we can mention gender, level of education, socioeconomic status, and bilingualism.

### 5.1. Gender

Gender differences in language abilities have been widely analyzed in the psychological and neuropsychological literature, with frequent statements that women achieve higher performance on several verbal tests (e.g., [84, 106, 107]), usually show faster language development [31, 108], and have a larger vocabulary, more accurate speech production, and greater fluency [109, 110]. It is important to mention, however, that the proportion of this variance explained by gender is usually small [111, 112] and that in some reports the language advantage favors boys rather than girls [113]. In addition to behavioral dissimilarities between males and females, sexual differences in white and gray matter volume and brain functioning have been well documented [114–116]. Moreover, gender effects have been described in the reduction of gray matter and the increase in the volume of white matter that occurs in brain development during childhood and adolescence [51, 117, 118]. For instance, de Bellis et al. [119] found a greater age-related decline in gray matter and a corresponding increase in white matter in boys compared to girls. Although girls also showed significant developmental changes, these modifications took place at a slower rate than that in boys. These gender differences in brain development have been corroborated by other authors [120].

### 5.2. Education and Socioeconomic Status

Language abilities have also been strongly correlated with socioeconomic status and levels of education [121, 122]. For example, parents from low socioeconomic households use more nonverbal than verbal strategies with their children [123], which results in slower language acquisition. The language used by people from low socioeconomic sectors has also been reported as less fluent, characterized by a simpler grammatical structure, and much more reliant on emotional than logical strategies [124]. Performance on phonemic fluency tests by illiterate people is extremely poor, and the data currently available suggest that fluency in illiterate individuals may reach only 3-4 words per minute, at least for Spanish and Greek, though this may vary by language [125–127]. Language repetition ability in illiterate individuals is equivalent to that of schooled literates as long as real, high-frequency words are presented; however, when pseudowords are used, discrepancies become apparent [125, 128, 129].

It seems then that formal education facilitates the development of language into a fully symbolic tool. However, language development is strongly dependent on cultural values as well. At least one study [130] has shown that rural children with little schooling performed better than schooled Indian or American children in coding and decoding culturally relevant objects, such as grains and seeds. Thus, children with no formal schooling were able to separate language symbols from their physical referents and then use them to communicate accurately, though their displays of this ability depended on the cultural relevance of the stimuli used [131].

Schooling appears to influence functional brain organization [132] (for a review see Ardila et al. [121]), and SES differences in the function and structure of certain language-supporting brain regions have been reported [133, 134].

### 5.3. Bilingualism

Another variable that may influence the effects of age on the brain's organization of language is the subject's experience with one or more languages. It has been shown, for example, that infants who grow up in bilingual environments may have different windows for perceptual narrowing by retaining greater sensitivity to nonnative contrasts that reached a less narrow end state than monolingual infants [135]. There is also evidence of differences in white matter between monolinguals and bilinguals. Luk et al. [136] used diffusion tensor imaging (DTI) and fMRI to measure white matter integrity and resting-state functional connectivity in a comparative study of monolingual and bilingual older adults. The latter showed higher white matter integrity mainly in the corpus callosum that extended into the bilateral superior longitudinal fasciculi, the right inferior frontal-occipital fasciculus, and the uncinate fasciculus. While bilingualism plays an important role at older ages, potentially protecting against age-associated cognitive decline, its effect is somewhat muted in adulthood [137, 138].

Also worth noting is the fact that the characteristics of language circuitry seem to be susceptible to the way in which bilinguals acquire the second language. Mohades et al. [139] obtained the mean fractional anisotropy (FA) for 4 major white matter pathways in 45 children aged 8–11, subdivided into 3 groups (15 simultaneous bilinguals, 15 sequential bilinguals, and 15 monolinguals). The 3 groups showed no significant differences in mean FA over the left arcuate fasciculus/superior longitudinal fasciculus or the fibers emerging from the anterior mid-body of the corpus callosum that are associated with the premotor and supplementary motor cortices. In simultaneous bilingual subjects, the left inferior frontooccipital fasciculus had higher mean FA values compared to monolinguals and sequential bilinguals, whereas comparisons of the bundle that arises from the anterior area of the corpus callosum and projects into the orbital lobe fibers yielded a significantly lower mean FA value in simultaneous bilingual subjects compared to monolinguals. In both cases, the FA values for sequential bilinguals were intermediate between those of the other two groups. To the best of our knowledge, this study provides the first evidence of bilingualism-related adaptations of white matter microstructure in the human brain.

There is also evidence for the plasticity of cortical gray matter in response to bilingualism. For example, Mechelli et al. [140] reported higher gray matter density in left inferior parietal regions in a group of Italian-English bilinguals relative to English monolinguals. The simultaneous use of two different languages has been seen to be associated with functional brain changes and different connectivity patterns. Future research will determine the bilingual variables associated with these connectivity changes.

## 6. Conclusions

This review has attempted to elucidate the typical development of language in relation to typical brain development and to reach some conclusions drawn by integrating research from the fields of neuropsychology and neuroimaging. Structural neuroimaging studies have shown a positive correlation between language tests and WM volume; that is, as WM increases in childhood, better performance on language tests is seen. In contrast, a negative correlation is observed between language test performance and GM volume in children, with decreased GM being associated with better performance. In senescence, there is a positive correlation between GM volume and language test performance.

Functional brain organization shows modifications with age, and these changes in brain dynamics are also associated with performance on language tasks. Brain activation during language tasks moves from bilateral (early in life) to unilateral (young adults) and then back to bilateral (senescence). Although data point to an asymmetrical distribution of language from birth, lateralization of language in the left hemisphere is modified by experience and, according to many authors, greater lateralization of language in the left hemisphere seems to be an index of maturation. Interestingly, older adults with bilateral activation achieve better language test performance.

There is a clear need for additional studies on several topics: first, well-elaborated models of neurocognitive development for individuals across the life span that are applicable to language development from childhood to senescence. Some neurocognitive models have already been proposed for older individuals, such as the vulnerability of anterior brain systems in aging [141, 142]; the brain reorganization hypothesis proposed in the HAROLD model; and the posterior anterior shift in aging [80, 143]. However, there is an evident need to continue advancing in this direction. A second area should focus on implementing longitudinal designs that combine neuroimaging and neuropsychological data from large sample groups at different levels of development, ideally spanning the entire age spectrum from childhood to senescence. Although some of the studies described in this review were longitudinal, most were of the cross-sectional type which limits the possibilities of generalizing their results. A third research area would involve using structural equation models (i.e., predictive models; see [144]) in studies of language development, as this would allow us to make better predictions of the influence of age in relation to other intervening variables, such as gender, years of schooling, SES, and language experience.

## Figures and Tables

**Table 1:** Acquisition of phonemes in English (adapted from Sander, [21]).

Phonemes	Age at which 50% produced the sound	Age at which 90% produced the sound
/p/, /m/, /h/, /n/, /w/	1 year	3 years
/b/	1 year	4 years
/k/, /g/, /d/	2 years	4 years
/t/, /ŋ/	2 years	6 years
/f/	2.5 years	4 years
/r/, /l/	3 years	6 years
/s/	3 years	8 years
/t*∫*/, /*∫*/	3.5 years	7 years
/z/	3.5 years	8 years
/j/	4 years	7 years
/v/	4 years	8 years
/*θ*/	4.5 years	7 years
/ð/	5 years	8 years
/ʒ/	6 years	8.5 years

**Table 2:** Acquisition of phonemes in Spanish (adapted from Bedore, [23]).

Phonemes	Age at which 50% produced the sound	Age at which 90% produced the sound
/p/, /b/, /t/	3 years	3.3 years
/m/, /n/, /k/	3 years	3.7 years
/ʝ/	3 years	3.9 years
/l/	3.3 years	3.9 years
/ʎ/	—	—
/f/	3 years	4.3 years
/t*∫*/	3 years	4.5 years
/d/, /g/	3.3 years	4.5 years
/r/	3.7 years	4.5 years
/x/	3.3 years	4.9 years
/ɲ/	3.7 years	4.9 years
/s/	3.3 years	5.5 years
/ɾ/	4.7 years	6 years

Note. The phoneme /ʎ/ is preserved in some areas of Spain and Latin America.

**Table 3:** Mean length of utterances in words (MLUw) and morphemes (MLUm) per age group (adapted from Rice et al. [39]).

Age range (in years)	MLUw	MLUm
2; 6–2; 11	2.91	3.23
3-3; 11	3.57	3.95
4-4; 11	4.19	4.66
5-5; 11	4.42	4.92
6-6; 11	4.63	5.14
7-7; 11	4.82	5.33
8-8; 11	5.03	5.59

Note. The scores presented by Rice et al. [39] were averaged for each year range.

**Table 4:** Verbal fluency means and (standard deviations) for children and adolescents.

Reference/category	*N*	Language	Age (years)										
			5-6	6-7	7-8	8-9	9-10	10-11	11-12	12-13	13-14	14-15	16-17
Crowe and Prescott [145]	155	English											
Animals			8.9 (3.0)		12.3 (4.6)		15.8 (4.0)						
Body parts			9.9 (4.2)		13.0 (5.1)		17.1 (4.5)						
Clothes			8.4 (2.8)		11.0 (3.9)		12.3 (4.4)						
Foods			8.1 (2.9)		11.9 (4.9)		14.3 (4.1)						
Plants			3.7 (2.7)		5.5 (2.5)		7.4 (3.7)						
Vehicles			5.3 (2.2)		8.3 (3.0)		9.7 (3.3)						
Filippetti and Allegri [146]	120	Spanish											
Animals					12.0 (3.3)		14.2 (3.6)						
Fruits					7.3 (1.9)		9.0 (2.3)						
/f/					4.9 (1.8)		7.2 (2.7)						
/a/					5.7 (2.6)		6.8 (2.5)						
/s/					5.1 (2.4)		6.7 (2.9)						
Kavé [56]	180	Hebrew											
Phonemic-bet					5.3 (1.8)		7.4 (2.1)		9.4 (3.4)		11.7 (3.8)	13.5 (3.9)	
Gimel					5.7 (2.5)		7.6 (2.3)		9.8 (3.1)		12.2 (3.5)	13.3 (3.6)	
Shin					6.5 (2.7)		7.2 (2.8)		10.3 (3.5)		11.0 (3.2)	13.8 (4.0)	
Animals, fruits and vegetables, vehicles					27.2 (9.2)		36.7 (10.2)		43.7 (10.4)		46.2 (10)	53 (10.3)	
Matute et al. [57]	171	Spanish											
Fruits			9.8 (3.6)		11.2 (2.7)		12.4 (2.6)		13.5 (2.9)		15.7 (3.1)		
/m/			5.8 (2.8)		7.2 (3.0)		8.6 (2.8)		9.3 (3.4)		12.5 (4.4)		
Sauzéon et al. [59]	140	English										15-16 yrs old	
FAS				4.5 (1.4)	6.3 (1.9)			7.6 (2.0)		9.1 (2.1)		10.6 (2.2)	
Fruit & supermarket				8.8 (2.2)	11.5 (2.5)			13.2 (3.0)		14.3 (2.5)		15.1 (2.3)	
Tallberg et al. [147]	130	Swedish	6 yrs old			9 yrs old			12 yrs old			15 yrs old	
FAS			8.8 (6.0)			16.0 (5.6)			27.3 (6.0)			30.1 (8.4)	
Animals			9.5 (3.2)			13.5 (4.0)			17.6 (4.4)			18.7 (3.9)	
Chan and Poon [148]	90	Chinese		7–11 yrs old					12–14 yrs old			15–18 yrs olds	
Animals				15.4 (3.2)					17.7 (4.2)			18.5 (3.9)	
Transportation				10.6 (3.1)					11.8 (3.4)			13.9 (4.3)	

**Table 5:** Mean and (standard deviations) for different verbal fluency tests by age group.

Reference	*N*	Language	Age group and task	Age groups						
Albert et al. [149]	80	English	Age group		30–39	50–59	60–69	70–80		
			FAS		49.2 (9.11)	46.1 (9.4)	45.3 (11.6)	39.7 (10.4)		

Auriacombe et al. [150]	1133	French	Age group				≤74	75–79	80–84	≥85
			Letter (L & P)				19.8 (7.7)	17.8 (7.5)	15.2 (6.5)	15 (6.1)
			Color & Animals				31.4 (7.6)	29.2 (6.9)	26.6 (6.6)	23.9 (6.2)

Bolla et al. [151]	478	English	Age group			55–69		70–79	80–94	
			Animals			19.6 (4.1)		18.8 (4.7)	18.3 (4.7)	
			Fruits			15.4 (3.2)		15.1 (3.5)	13.5 (3.3)	
			Vegetables			14.2 (3.1)		14.2 (3.4)	12.7 (3.5)	
			FAS			48.4 (11.7)		47.8 (13.2)	44.8 (12.9)	

Chan and Poon [148]	226	Chinese	Age groups	19–30	31–40	41–50	51–60	61–70	71–80	
			Animals	19.6 (4.0)	16.8 (4.6)	16.3 (3.4)	15.5 (5.4)	15.8 (4.3)	13.6 (3.7)	
			Transportation	14.1 (3.3)	13.0 (3.0)	12.8 (3.6)	9.4 (3.3)	10.3 (3.5)	9.1 (3.2)	

Foldi et al. [152]	73	English	Age groups	18–39		40–59	60–74	75–88		
			Animals	21.4 (3.7)		21.1 (5.0)	17.2 (5.1)	15.1 (4.9)		
			Letter M	13.3 (3.3)		13.1 (3.5)	10.7 (3.5)	10.8 (4.7)		

Gordon and Kindred [153]	56	English	Age groups		30–49		50–69	70–90		
			Animals		27.8		24.5	21.5		
			FAS		16.7		15.5	13.6		

Kavé [154]	369	Hebrew	Age groups	18–30	31–50	51–70		71–85		
			Bet	13.5 (3.7)	11.8 (3.6)	11.1 (3.5)		9.5 (2.9)		
			Gimel	14.4 (3.5)	12.9 (3.5)	12.1 (3.1)		10.4 (3.6)		
			Shin	15.2 (4.0)	14.2 (3.9)	13.3 (3.7)		10.9 (3.2)		
			Animals	24.8 (5.2)	22.8 (5.5)	19.8 (5.1)		17.9 (4.7)		
			Fruits and vegetables	23.2 (4.5)	23.1 (4.6)	21.1 (4.3)		18.0 (4.4)		
			Vehicles	15.1 (3.4)	15.4 (3.9)	13.6 (3.6)		11.7 (3.0)		

Khalil [155]	215	Arabic	Age groups	17–29	30–39	40–59				
			Phonemic WRG	29.2 (5.0)	29.8 (4.7)	24.1 (3.0)				
			Animals	16.6 (3.3)	17.8 (2.5)	14.9 (3.4)				

Mejia et al. [156]	60	Spanish	Age groups			55–70		71–85		
			FAS			10.2 (8.3)		8.0 (2.9)		
			Animals and fruits			14.2 (2.2)		11.6 (3.0)		

Sauzéon et al. [157]	40	French	Age groups	18–23		65–75				
			Phonemic FS	29.6 (3.77)		29.2 (4.27)				
			Supermarket	22.6 (2.92)		24.3 (2.82)				

Ryu et al. [158]	3025	Korean	Age			60–69	70–74	74–79	80–84	≥85
						13.6 (4.0)	13.3 (3.9)	12.9 (3.8)	12.4 (3.7)	11.9 (3.7)

Stokholm et al. [159]	100	Danish	Age group				60–87			
			Animals				21.3 (4.8)			
			Supermarket				23.6 (5.8)			
			S-words				13.7 (5.8)			
			Action word				13.3 (4.7)			

Troyer et al. [160]	95	English	Age group	18–35	60–89					
			FAS	41.9 (11.5)	41.3 (10.9)					
			Animals	21.8 (5.7)	17.8 (4.2)					

Schmitter-Edgecombe et al. [161]	78	English	Age group	18–22			58–74	75–93		
			FAS	43.3 (11.4)			41.4 (9.8)	37.4 (8.5)		
			Animals	20.2 (3.7)			21.1 (4.8)	17.1 (4.7)		

Tallberg et al. [147]	165	Swedish	Age group	16–29		30–64		65–89		
			FAS	40.0 (10.7)		45.7 (11.7)		40.9 (12.6)		
			Animals	24.7 (4.6)		26.0 (5.9)		19.2 (5.7)		

Wecker et al. [162]	719	English	Age group	20–29	30–39	40–49	50–59	60–69	70–79	80–89
			Animals	20.0 (4.5)	20.5 (4.2)	19.8 (4.3)	18.3 (4.3)	17.1 (4.2)	16.3 (3.8)	14.6 (3.8)

**Table 6:** Summary of main findings of brain organization of language using neuroimaging techniques from infancy to adulthood.

Author	*N*	Age	Main findings	Language test	Technique
Leroy et al. [36]	14	1–4 months	The ventral superior temporal sulcus (STS) is less mature than the inferior frontal area. A significant difference of maturation in the STS favors the right side.	None	MRI

Dehaene-Lambertz et al. [11]	20	3 months	Left-lateralized brain regions (the superior temporal and angular gyri) were already active in infants.	Oral speech stimuli	fMRI

Pujol et al. [35]	100	0–3 yrs.	Changes in the volume of myelinated WM began in the sensorimotor WM and the Heschl gyrus and extended to language-related areas. Both comprehension and production regions showed a very similar myelination course.	None	MRI

Su et al. [34]	241 25	0–429 wks. 14-to-83 years	Higher cortical areas (Broca and Wernicke) matured later than the primary cortical areas, while the arcuate fasciculus matured last.	None	MRI

Szaflarski et al. [67]	30	5, 6 & 7 yrs.	With increasing age, there is progressive participation of the inferior/middle frontal, middle temporal, and angular gyri of the left hemisphere and the lingual and inferior temporal gyri of the right hemisphere	Verb generation task	fMRI

Ressel et al. [69]	22	7–16 yrs.	Significant increase of left hemisphere lateralization as a function of age was observed for both tasks.	Verb generation task and vowel-identification	MEG

Brown et al. [66]	95	7–32 yrs.	Systematic increases and decreases in cortical activity over age, by region. Age-related increases in activity were primarily in the left frontal and left parietal cortex. Decreases attenuated with age and were found across a broader neuroanatomical range, containing earlier processing regions such as the bilateral extrastriate cortex.	Word generation	fMRI

Kadis et al. [42]	28	5–19 yrs.	Positive correlation between left hemisphere lateralization during this language task and age.	Verb generation task	MEG

Friederici et al. [13]	15 children 16 adults	5–7 yrs. 26–30 yrs.	While adults display a network clearly lateralized in the left hemisphere underlying sentence processing, 6-year-old children demonstrate stronger inter-hemispheric connectivity.	Sentence grammar identification	fMRI

Holland et al. [58]	17	7–19 yrs.	Significant association between hemisphere lateralization and age was found. Although most subjects at all ages showed left hemisphere dominance for this task, the degree of lateralization increased with age.	Verb generation task	fMRI

Lidzba et al. [55]	36	6–24 yrs.	Language comprehension was associated with more focal activation with age in the bilateral superior temporal gyri with no increases of lateralization with age. The language production task showed an increase with age both in focus and lateralization.	Beep stories task (language comprehension) and Vowel identification task (language production task)	fMRI

Schlösser et al. [97]	12	22–26 yrs.	Activation in the left prefrontal cortex and right cerebellum.	Verbal fluency task	fMRI

Meinzer et al. [100]	16 16	20–33 yrs. 66–88 yrs.	Performance during the phonemic task was equivalent for both age groups and mirrored by strongly left-lateralized (frontal) activity patterns. The decline in performance during the semantic task in the older group was complemented with additional right (inferior and middle) frontal activity, which was negatively correlated with performance.	Semantic and phonemic fluency tasks	fMRI

Nuñez et al. [41]	19	7.2–15.8 yrs.	Increased activation in the left and decreased activation in the right inferior front gyrus (with surge of cortical thickness in the right) was associated with increased syntactic proficiency. A maturational shift towards decreased involvement of the right IFG for syntactic processing is found.	Sentence comprehension and judgment task	fMRI

Obler et al. [96]	24	56–79 yrs.	Older adults with better naming skills could rely on right-hemisphere perisylvian and mid-frontal regions and pathways, in conjunction with left-hemisphere perisylvian and mid-frontal regions, to achieve success.	Boston Naming Test and Action Naming Test	MRI and DTI

Abrahams et al. [98]	18	39–76 yrs.	Verbal fluency was associated with activation in the middle frontal gyrus (BA 46 and 9), the anterior cingulate gyrus, and the inferior frontal gyrus (area 44 and 45). Confrontation naming activated areas of the temporooccipital cortices (areas 18, 19, and 37) and the inferior frontal gyrus.	Verbal fluency and confrontation	fMRI

Note. MRI = magnetic Resonance Imaging; fMRI = functional; MEG = magnetoencephalography; DIT = diffusion tensor imaging.

## References

[1] Martinet A. (1960). *Éléments de Linguistique Générale*.

[2] Crystal D. (2011). *Dictionary of Linguistics and Phonetics*.

[3] Ardila A. (2011). There are two different language systems in the brain. *Journal of Behavioral and Brain Science*.

[4] Bickerton D. (2007). Language evolution: a brief guide for linguists. *Lingua*.

[5] Chomsky N. (1980). *Rules and Representations*.

[6] Ardila A. (2012). Interaction between lexical and grammatical language systems in the brain. *Physics of Life Reviews*.

[7] Hervé P.-Y., Zago L., Petit L., Mazoyer B., Tzourio-Mazoyer N. (2013). Trends in Cognitive Sciences. *Trends in cognitive sciences*.

[8] DeCasper A. J., Fifer W. P. (1980). Of human bonding: newborns prefer their mothers' voices. *Science*.

[9] Slater A. (1998). *Perceptual Development: Visual, Auditory, and Speech Perception in Infancy*.

[10] Hiscock M., Kinsbourne M., Davidson R. J., Hugdahl K. (1995). Phylogeny and ontogeny of cerebral lateralization. *Brain Asymmetry*.

[11] Dehaene-Lambertz G., Dehaene S., Hertz-Pannier L. (2002). Functional neuroimaging of speech perception in infants. *Science*.

[12] Dick F., Leech R., Richardson F., Reed J., Warner-Rogers J. (2008). The neuropsychology of language development. *Child Neuropsychology : Concepts, Theory and Practice*.

[13] Friederici A. D., Brauer J., Lohmann G. (2011). Maturation of the language network: from inter- to intrahemispheric connectivities. *PLoS ONE*.

[14] Everts R., Lidzba K., Wilke M. (2009). Strengthening of laterality of verbal and visuospatial functions during childhood and adolescence. *Human Brain Mapping*.

[15] Blumstein S. E., Amso D. (2013). Dynamic functional organization of language: insights from functional neuroimaging. *Perspectives on Psychological Science*.

[16] Kelly D. J., Quinn P. C., Slater A. M., Lee K., Ge L., Pascalis O. (2007). The other-race effect develops during infancy: evidence of perceptual narrowing. *Psychological Science*.

[17] Kuhl P. K., Conboy B. T., Coffey-Corina S., Padden D., Rivera-Gaxiola M., Nelson T. (2008). Phonetic learning as a pathway to language: new data and native language magnet theory expanded (NLM-e). *Philosophical Transactions of the Royal Society B: Biological Sciences*.

[18] Werker J. F., Tees R. C. (2002). Cross-language speech perception: evidence for perceptual reorganization during the first year of life. *Infant Behavior and Development*.

[19] Faulkner R. L., Low L. K., Cheng H.-J. (2006). Axon Pruning in the developing vertebrate hippocampus. *Developmental Neuroscience*.

[20] Lewkowicz D. J., Ghazanfar A. A., Kapur N. (2011). Paradoxical psychological functioning in early childhood development. *The Paradoxical Brain*.

[21] Sander E. K. (1972). When are speech sounds learned?. *Journal of Speech and Hearing Disorders*.

[22] Hoff E. (2009). *Language Development*.

[23] Bedore L., Taylor O. L., Leonard L. (1999). The acquisition of Spanish. *Language Acquisition Across North America: Cross-Cultural and Cross-Linguistic Perspectives*.

[24] Smit A. B., Hand L., Freilinger J. J., Bernthal J. E., Bird A. (1990). The Iowa articulation norms project and its Nebraska replication. *Journal of Speech and Hearing Disorders*.

[25] Vihman M. M. (1996). *Phonological Development. The Origins of Language in the Child*.

[26] Gibson K. R., Petersen A. C. (2010). *Brain Maturation and Cognitive Development*.

[27] Vigneau M., Beaucousin V., Hervé P. Y. (2006). Meta-analyzing left hemisphere language areas: phonology, semantics, and sentence processing. *NeuroImage*.

[28] Fenson L., Bates E., Dale P., Goodman J., Reznick J. S., Thal D. (2000). Measuring variability in early child language: don't shoot the messenger. *Child Development*.

[29] Jackson-Maldonado D., Marchman V. A., Fernald L. C. H. (2013). Short-form versions of the Spanish MacArthur-Bates communicative development inventories. *Applied Psycholinguistics*.

[30] Bates E., Goodman J. C. (1997). On the inseparability of grammar and the lexicon: evidence form acquisition, aphasia and real-time processing. *Language and Cognitive Processes*.

[31] Fenson L., Dale P. S., Reznick J. S., Bates E., Thal D. J., Pethick S. J. (1994). Variability in early communicative development. *Monographs of the Society for Research in Child Development*.

[32] Lorraine S. (2008). *Vocabulary Development: Super Duper Handouts Number 149*.

[33] Courchesne E., Pierce K. (2005). Brain overgrowth in autism during a critical time in development: Implications for frontal pyramidal neuron and interneuron development and connectivity. *International Journal of Developmental Neuroscience*.

[34] Su P., Kuan C. C., Kaga K., Sano M., Mima K. (2008). Myelination progression in language-correlated regions in brain of normal children determined by quantitative MRI assessment. *International Journal of Pediatric Otorhinolaryngology*.

[35] Pujol J., Soriano-Mas C., Ortiz H., Sebastián-Gallés N., Losilla J. M., Deus J. (2006). Myelination of language-related areas in the developing brain. *Neurology*.

[36] Leroy F., Glasel H., Dubois J. (2011). Early maturation of the linguistic dorsal pathway in human infants. *The Journal of Neuroscience*.

[37] Gervain J., Macagno F., Cogoi S., Peña M., Mehler J. (2008). The neonate brain detects speech structure. *Proceedings of the National Academy of Sciences of the United States of America*.

[38] Tomasello M. (2003). *Constructing a Language: A Usage-Based Theory of Language Acquisition*.

[39] Rice M. L., Smolik F., Perpich D., Thompson T., Rytting N., Blossom M. (2010). Mean length of utterance levels in 6-month intervals for children 3 to 9 years with and without language impairments. *Journal of Speech, Language, and Hearing Research*.

[40] Brassard M. R., Boehm A. E. (2008). *Preschool Assessment: Principles and Practices*.

[41] Nuñez S. C., Dapretto M., Katzir T. (2011). fMRI of syntactic processing in typically developing children: structural correlates in the inferior frontal gyrus. *Developmental Cognitive Neuroscience*.

[42] Kadis D. S., Pang E. W., Mills T., Taylor M. J., McAndrews M. P., Smith M. L. (2011). Characterizing the normal developmental trajectory of expressive language lateralization using magnetoencephalography. *Journal of the International Neuropsychological Society*.

[43] Riva D., Nichelli F., Devoti M. (2000). Developmental aspects of verbal fluency and confrontation naming in children. *Brain and Language*.

[44] Reed J., Warner-Rogers J. (2008). *Child Neuropsychology: Concepts, Theory, and Practice*.

[45] Lenroot R. K., Giedd J. N. (2006). Brain development in children and adolescents: insights from anatomical magnetic resonance imaging. *Neuroscience & Biobehavioral Reviews*.

[46] Giedd J. N., Rapoport J. L. (2010). Structural MRI of pediatric brain development: what have we learned and where are we going?. *Neuron*.

[47] Paus T., Zijdenbos A., Worsley K. (1999). Structural maturation of neural pathways in children and adolescents: in vivo study. *Science*.

[48] Wilke M., Lidzba K., Krägeloh-Mann I. (2009). Combined functional and causal connectivity analyses of language networks in children: a feasibility study. *Brain and Language*.

[49] Kochunov P., Williamson D. E., Lancaster J. (2012). Fractional anisotropy of water diffusion in cerebral white matter across the lifespan. *Neurobiology of Aging*.

[50] Toga A. W., Thompson P. M., Sowell E. R. (2006). Mapping brain maturation. *Trends in Neurosciences*.

[51] Sowell E. R., Trauner D. A., Gamst A., Jernigan T. L. (2002). Development of cortical and subcortical brain structures in childhood and adolescence: a structural MRI study. *Developmental Medicine & Child Neurology*.

[52] Giorgio A., Watkins K. E., Douaud G. (2008). Changes in white matter microstructure during adolescence. *NeuroImage*.

[53] Bava S., Thayer R., Jacobus J., Ward M., Jernigan T. L., Tapert S. F. (2010). Longitudinal characterization of white matter maturation during adolescence. *Brain Research*.

[54] Schmithorst V. J., Yuan W. (2010). White matter development during adolescence as shown by diffusion MRI. *Brain and Cognition*.

[55] Lidzba K., Schwilling E., Grodd W., Krägeloh-Mann I., Wilke M. (2011). Language comprehension vs. language production: age effects on fMRI activation. *Brain and Language*.

[56] Kavé G. (2006). The development of naming and word fluency: evidence from Hebrew-speaking children between ages 8 and 17. *Developmental Neuropsychology*.

[57] Matute E., Rosselli M., Ardila A., Morales G. (2004). Verbal and non-verbal fluency in six to 15-year-old Spanish-speaking children. *Developmental Neuropsychology*.

[58] Holland S. K., Plante E., Byars A. W., Strawsburg R. H., Schmithorst V. J., Ball W. S. (2001). Normal fMRI brain activation patterns in children performing a verb generation task. *NeuroImage*.

[59] Sauzéon H., Lestage P., Raboutet C., N'Kaoua B., Claverie B. (2004). Verbal fluency output in children aged 7–16 as a function of the production criterion: qualitative analysis of clustering, switching processes, and semantic network exploitation. *Brain and Language*.

[60] Koren R., Kofman O., Berger A. (2005). Analysis of word clustering in verbal fluency of school-aged children. *Archives of Clinical Neuropsychology*.

[61] Lenroot R. K., Gogtay N., Greenstein D. K. (2007). Sexual dimorphism of brain developmental trajectories during childhood and adolescence. *NeuroImage*.

[62] Giedd J. N., Blumenthal J., Jeffries N. O. (1999). Brain development during childhood and adolescence: a longitudinal MRI study. *Nature Neuroscience*.

[63] Giedd J. N., Lalonde F. M., Celano M. J. (2009). Anatomical brain magnetic resonance imaging of typically developing children and adolescents. *Journal of the American Academy of Child and Adolescent Psychiatry*.

[64] Fields R. D., Stevens-Graham B. (2002). New insights into neuron-glia communication. *Science*.

[65] Lebel C., Beaulieu C. (2011). Longitudinal development of human brain wiring continues from childhood into adulthood. *The Journal of Neuroscience*.

[66] Brown T. T., Lugar H. M., Coalson R. S., Miezin F. M., Petersen S. E., Schlaggar B. L. (2005). Developmental changes in human cerebral functional organization for word generation. *Cerebral Cortex*.

[67] Szaflarski J. P., Schmithorst V. J., Altaye M. (2006). A longitudinal functional magnetic resonance imaging study of language development in children 5 to 11 years old. *Annals of Neurology*.

[68] Desmond J. E., Sum J. M., Wagner A. D. (1995). Functional MRI measurement of language Lateralization in Wada-tested patients. *Brain*.

[69] Ressel V., Wilke M., Lidzba K., Lutzenberger W., Krägeloh-Mann I. (2008). Increases in language lateralization in normal children as observed using magnetoencephalography. *Brain and Language*.

[70] Allen L. S., Richey M. F., Chai Y. M., Gorski R. A. (1991). Sex differences in the corpus callosum of the living human being. *The Journal of Neuroscience*.

[71] Thompson P. M., Gledd J. N., Woods R. P., MacDonald D., Evans A. C., Toga A. W. (2000). Growth patterns in the developing brain detected by using continuum mechanical tensor maps. *Nature*.

[72] Westerhausen R., Luders E., Specht K. (2011). Structural and functional reorganization of the corpus callosum between the age of 6 and 8 years. *Cerebral Cortex*.

[73] Brown A. S. (1991). A review of the tip-of-the-tongue experience. *Psychological Bulletin*.

[74] Ardila A. (2007). Normal aging increases cognitive heterogeneity. *Archives of Clinical Neuropsychology*.

[75] Wechsler D. (1997). *WAIS-III: Administration and Scoring Manual*.

[76] Verhaegen C., Poncelet M. (2013). Changes in naming and semantic abilities with aging from 50 to 90 years. *Journal of the International Neuropsychological Society*.

[77] Wingfield A., Grossman M. (2006). Language and the aging brain: patterns of neural compensation revealed by functional brain imaging. *Journal of Neurophysiology*.

[78] Szaflarski J. P., Holland S. K., Schmithorst V. J., Byars A. W. (2006). fMRI study of language lateralization in children and adults. *Human Brain Mapping*.

[79] Cabeza R., Anderson N. D., Locantore J. K., McIntosh A. R. (2002). Aging gracefully: compensatory brain activity in high-performing older adults. *NeuroImage*.

[80] Cabeza R. (2002). Hemispheric asymmetry reduction in older adults: the HAROLD model. *Psychology and Aging*.

[81] Dennis N. A., Hayes S. M., Prince S. E., Madden D. J., Huettel S. A., Cabeza R. (2008). Effects of aging on the neural correlates of successful item and source memory encoding. *Journal of Experimental Psychology: Learning Memory and Cognition*.

[82] Cabeza R., Dennis N. A., Stuss D. T., Knight R. T. (2012). Frontal lobes and aging: deterioration and compensation. *Principles of Frontal Lobe Function*.

[83] Maguire E. A., Frith C. D. (2003). Aging affects the engagement of the hippocampus during autobiographical memory retrieval. *Brain*.

[84] Ardila A., Rosselli M. (1989). Neuropsychological characteristics of normal aging. *Developmental Neuropsychology*.

[85] Burke D., Worthley J., Martin J., Gruneberg M. M., Morris P. E., Sykes J. N. (1988). I'll never forget what's-her-name: aging and the tip of the tongue experience in everyday life. *Practical Aspects of Memory: Current Research and Theory*.

[86] Mackay A. J., Connor L. T., Albert M. L., Obler L. K. (2002). Noun and verb retrieval in healthy aging. *Journal of the International Neuropsychological Society*.

[87] LaBarge E., Edwards D., Knesevich J. W. (1986). Performance of normal elderly on the Boston naming test. *Brain and Language*.

[88] Villardita C., Cultrera S., Cupone V., Mejia R. (1985). Neuropsychological test performance and normal aging. *Archives of Gerontology and Geriatrics*.

[89] Kent P. S., Luszcz M. A. (2002). A review of the Boston Naming Test and multiple-occasion normative data for older adults on 15-item versions. *The Clinical Neuropsychologist*.

[90] Connor L. T., Spiro A., Obler L. K., Albert M. L. (2004). Change in object naming ability during adulthood. *Journal of Gerontology: Psychological Sciences*.

[91] Zec R. F., Burkett N. R., Markwell S. J., Larsen D. L. (2007). A cross-sectional study of the effects of age, education, and gender on the Boston Naming Test. *Clinical Neuropsychologist*.

[92] Weintraub S., Powell D. H., Whitla D. K. (1994). Successful cognitive aging: individual differences among physicians on a computerized test of mental state. *The Journal of Geriatric Psychiatry*.

[93] Au R., Joung P., Nicholas M., Obler L. K., Kass R., Albert M. L. (1995). Naming ability across the adult life span. *Aging and Cognition*.

[94] Lezak M. D., Howieson D. B., Bigler E. D., Tranel D. (2012). *Neuropsychological Assessment*.

[95] Hurks P. P. M., Schrans D., Meijs C., Wassenberg R., Feron F. J. M., Jolles J. (2010). Developmental changes in semantic verbal fluency: analyses of word productivity as a function of time, clustering, and switching. *Child Neuropsychology*.

[96] Obler L. K., Rykhlevskaia E., Schnyer D. (2010). Bilateral brain regions associated with naming in older adults. *Brain and Language*.

[97] Schlösser R., Hutchinson M., Joseffer S. (1998). Functional magnetic resonance imaging of human brain activity in a verbal fluency task. *Journal of Neurology Neurosurgery and Psychiatry*.

[98] Abrahams S., Goldstein L. H., Simmons A. (2003). Functional magnetic resonance imaging of verbal fluency and confrontation naming using compressed image acquisition to permit overt responses. *Human Brain Mapping*.

[99] Amunts K., Schleicher A., Zilles K. (2004). Outstanding language competence and cytoarchitecture in Broca's speech region. *Brain and Language*.

[100] Meinzer M., Flaisch T., Wilser L. (2009). Neural signatures of semantic and phonemic fluency in young and old adults. *Journal of Cognitive Neuroscience*.

[101] Bernal B., Perdomo J. http://www.fmriconsulting.com/brodmann/Introduction.html.

[102] Benson D. F., Ardila A. (1996). *Aphasia: A Clinical Perspective*.

[103] Dronkers N. F. (1996). A new brain region for coordinating speech articulation. *Nature*.

[104] Berthier M. (1999). *Transcortical Aphasias*.

[105] Fedorenko E., Behr M. K., Kanwisher N. (2011). Functional specificity for high-level linguistic processing in the human brain. *Proceedings of the National Academy of Sciences of the United States of America*.

[106] Burton L. A., Henninger D., Hafetz J. (2005). Gender differences in relations of mental rotation, verbal fluency, and SAT scores to finger length ratios as hormonal indexes. *Developmental Neuropsychology*.

[107] Weiss E. M., Kemmler G., Deisenhammer E. A., Fleischhacker W. W., Delazer M. (2003). Sex differences in cognitive functions. *Personality and Individual Differences*.

[108] Berglund E., Eriksson M., Westerlund M. (2005). Communicative skills in relation to gender, birth order, childcare and socioeconomic status in 18-month-old children. *Scandinavian Journal of Psychology*.

[109] Pinker S. (2007). *The Stuff of Thought*.

[110] Sommer I. E., Aleman A., Somers M., Boks M. P., Kahn R. S. (2008). Sex differences in handedness, asymmetry of the planum temporale and functional language lateralization. *Brain Research*.

[111] Hyde J. S., Linn M. C. (1988). Gender differences in verbal ability: a meta-analysis. *Psychological Bulletin*.

[112] Wallentin M. (2009). Putative sex differences in verbal abilities and language cortex: a critical review. *Brain and Language*.

[113] Ardila A., Rosselli M., Matute E., Inozemtseva O. (2011). Gender differences in cognitive development. *Developmental Psychology*.

[114] Gur R. C., Turetsky B. I., Matsui M. (1999). Sex differences in brain gray and white matter in healthy young adults: correlations with cognitive performance. *The Journal of Neuroscience*.

[115] Kanaan R. A., Allin M., Picchioni M. (2012). Gender differences in white matter microstructure. *PloS ONE*.

[116] Tian L., Wang J., Yan C., He Y. (2011). Hemisphere- and gender-related differences in small-world brain networks: a resting-state functional MRI study. *NeuroImage*.

[117] Hua X., Leow A. D., Levitt J. G., Caplan R., Thompson P. M., Toga A. W. (2009). Detecting brain growth patterns in normal children using tensor-based morphometry. *Human Brain Mapping*.

[118] Sowell E. R., Thompson P. M., Holmes C. J., Batth R., Jernigan T. L., Toga A. W. (1999). Localizing age-related changes in brain structure between childhood and adolescence using statistical parametric mapping. *NeuroImage*.

[119] de Bellis M. D., Keshavan M. S., Beers S. R. (2001). Sex differences in brain maturation during childhood and adolescence. *Cerebral Cortex*.

[120] Wilke M., Krägeloh-Mann I., Holland S. K. (2007). Global and local development of gray and white matter volume in normal children and adolescents. *Experimental Brain Research*.

[121] Ardila A., Bertolucci P. H., Braga L. W. (2010). Illiteracy: the neuropsychology of cognition without reading. *Archives of Clinical Neuropsychology*.

[122] Hoff E., Bornstein M. H., Bradley R. H. (2003). Causes and consequences of SES-related differences in parent-to-child speech. *Socioeconomic Status, Parenting and Child Development*.

[123] Robinson W. P., Huxley R., Ingram E. (1974). Social factors and language development in primary school children. *Language Acquisition: Models and Methods*.

[124] Bernstein B., Huxley R., Ingram E. (1974). Language and roles. *Language Acquisition: Models and Methods*.

[125] Kosmidis M. H., Tsapkini K., Folia V., Vlahou C. H., Kiosseoglou G. (2004). Semantic and phonological processing in illiteracy. *Journal of the International Neuropsychological Society*.

[126] Ostrosky-Solís F., Ardila A., Rosselli M. (1999). NEUROPSI: a brief neuropsychological test battery in Spanish with norms by age and educational level. *Journal of the International Neuropsychological Society*.

[127] Ostrosky-Solís F., García M. A., Pérez M. (2004). Can learning to read and write change the brain organization? An electrophysiological study. *International Journal of Psychology*.

[128] Petersson K. M., Reis A., Ingvar M. (2001). Cognitive processing in literate and illiterate subjects: a review of some recent behavioral and functional neuroimaging data. *Scandinavian Journal of Psychology*.

[129] Reis A., Castro-Caldas A. (1997). Illiteracy: a cause for biased cognitive development. *Journal of the International Neuropsychological Society*.

[130] Lantz D. (1979). A cross–cultural comparison of communication abilities: Some effects of age, schooling and culture. *International Journal of Psychology*.

[131] Mussen P., Laboratory of Comparative Human Cognition (1983). Culture and cognitive development. *Handbook of Child Psychology: History, Theories and Methods*.

[132] Castro-Caldas A., Peterson K. M., Reis A., Askelof S., Ingvar M. (1998). Differences in inter-hemispheric interactions related to literacy, assessed by PET. *Neurology*.

[133] Noble K. G., Wolmetz M. E., Ochs L. G., Farah M. J., McCandliss B. D. (2006). Brain-behavior relationships in reading acquisition are modulated by socioeconomic factors. *Developmental Science*.

[134] Raizada R. D. S., Richards T. L., Meltzoff A., Kuhl P. K. (2008). Socioeconomic status predicts hemispheric specialisation of the left inferior frontal gyrus in young children. *NeuroImage*.

[135] Byers-Heinlein K., Fennell C. T. (2014). Perceptual narrowing in the context of increased variation: insights from bilingual infants. *Developmental Psychobiology*.

[136] Luk G., Bialystok E., Craik F. I. M., Grady C. L. (2011). Lifelong bilingualism maintains white matter integrity in older adults. *Journal of Neuroscience*.

[137] Bialystok E., Craik F. I. M., Freedman M. (2007). Bilingualism as a protection against the onset of symptoms of dementia. *Neuropsychologia*.

[138] Salvatierra J., Rosselli M. (2011). The effect of bilingualism and age on inhibitory control. *International Journal of Bilingualism*.

[139] Mohades S. G., Struys E., van Schuerbeek P., Mondt K., van de Craen P., Luypaert R. (2012). DTI reveals structural differences in white matter tracts between bilingual and monolingual children. *Brain Research*.

[140] Mechelli A., Crinion J. T., Noppeney U. (2004). Neurolinguistics: structural plasticity in the bilingual brain. *Nature*.

[141] Head D., Buckner R. L., Shimony J. S. (2004). Differential vulnerability of anterior white matter in non-demented aging with minimal acceleration in dementia of the Alzheimer type: evidence from diffusion tensor imaging. *Cerebral Cortex*.

[142] Salthouse T. A. (1996). The processing speed theory of adult age differences in cognition. *Psychological Review*.

[143] Davis S. W., Dennis N. A., Daselaar S. M., Fleck M. S., Cabeza R. (2008). Que PASA? The posterior-anterior shift in aging. *Cerebral Cortex*.

[144] Charlton R. A., Landau S., Schiavone F. (2008). A structural equation modeling investigation of age-related variance in executive function and DTI measured white matter damage. *Neurobiology of Aging*.

[145] Crowe S. J., Prescott T. J. (2003). Continuity and change in the development of category structure: insights from the semantic fluency task. *International Journal of Behavioral Development*.

[146] Filippetti V. A., Allegri R. F. (2011). Verbal fluency in Spanish-speaking children: analysis model according to task type, clustering, and switching strategies and performance over time. *Clinical Neuropsychologist*.

[147] Tallberg I. M., Ivachova E., Jones Tinghag K., Östberg P. (2008). Swedish norms for word fluency tests: FAS, animals and verbs. *Scandinavian Journal of Psychology*.

[148] Chan A. S., Poon M. W. (1999). Performance of 7- to 95-year-old individuals in a Chinese version of the category fluency test. *Journal of the International Neuropsychological Society*.

[149] Albert M. S., Heller H. S., Milberg W. (1988). Changes in naming ability with age. *Psychology and Aging*.

[150] Auriacombe S., Fabrigoule C., Lafont S., Amieva H., Jacqmin-Gadda H., Dartigues J. F. (2001). Letter and category fluency in normal elderly participants: a population-based study. *Aging, Neuropsychology, and Cognition*.

[151] Bolla K. I., Gray S., Resnick S. M., Galante R., Kawas C. (1998). Category and letter fluency in highly educated older adults. *Clinical Neuropsychologist*.

[152] Foldi N. S., Helm-Estabrooks N., Redfield J., Nickel D. G. (2003). Perseveration in normal aging: a comparison of perseveration rates on design fluency and verbal generative tasks. *Aging, Neuropsychology, and Cognition*.

[153] Gordon J. K., Kindred N. K. (2011). Word retrieval in ageing: an exploration of the task constraint hypothesis. *Aphasiology*.

[154] Kavé G. (2005). Phonemic fluency, semantic fluency, and difference scores: normative data for adult Hebrew speakers. *Journal of Clinical and Experimental Neuropsychology*.

[155] Khalil M. S. (2010). Preliminary Arabic normative data of neuropsychological tests: the verbal and design fluency. *Journal of Clinical and Experimental Neuropsychology*.

[156] Mejia S., Pineda D., Alvarez L. M., Ardila A. (1998). Individual differences in memory and executive function abilities during normal aging. *International Journal of Neuroscience*.

[157] Sauzéon H., Raboutet C., Rodrigues J. (2011). Verbal knowledge as a compensation determinant of adult age differences in verbal fluency tasks over time. *Journal of Adult Development*.

[158] Ryu S.-H., Kim K. W., Kim S. (2012). Normative study of the category fluency test (CFT) from nationwide data on community-dwelling elderly in Korea. *Archives of Gerontology and Geriatrics*.

[159] Stokholm J., Jørgensen K., Vogel A. (2013). Performances on five verbal fluency tests in a healthy, elderly Danish sample. *Aging, Neuropsychology, and Cognition*.

[160] Troyer A. K., Moscovitch M., Winocur G. (1997). Clustering and switching as two components of verbal fluency: evidence from younger and older healthy adults. *Neuropsychology*.

[161] Schmitter-Edgecombe M., Vesneski M., Jones D. W. R. (2000). Aging and word-finding: a comparison of spontaneous and constrained naming tests. *Archives of Clinical Neuropsychology*.

[162] Wecker N. S., Kramer J. H., Hallam B. J., Delis D. C. (2005). Mental flexibility: age effects on switching. *Neuropsychology*.

